# Intensity-Dependent Changes in Quantified Resting Cerebral Perfusion With Multiple Sessions of Transcranial DC Stimulation

**DOI:** 10.3389/fnhum.2021.679977

**Published:** 2021-08-12

**Authors:** Matthew S. Sherwood, Lindsey McIntire, Aaron T. Madaris, Kamin Kim, Charan Ranganath, R. Andy McKinley

**Affiliations:** ^1^Science & Space, KBR Inc., Beavercreek, OH, United States; ^2^Infoscitex, Inc., Beavercreek, OH, United States; ^3^Department of Biomedical, Industrial and Human Factors Engineering, Wright State University, Dayton, OH, United States; ^4^Department of Psychology, University of California, Davis, Davis, CA, United States; ^5^Center for Neuroscience, University of California, Davis, Davis, CA, United States; ^6^Air Force Research Laboratory, Wright-Patterson AFB, Dayton, OH, United States

**Keywords:** MRI, arterial spin labeling, cerebral perfusion, neuromodulation, transcranial DC stimulation, prefrontal cortex, locus coeruleus

## Abstract

Transcranial direct current stimulation (tDCS) to the left prefrontal cortex has been shown to produce broad behavioral effects including enhanced learning and vigilance. Still, the neural mechanisms underlying such effects are not fully understood. Furthermore, the neural underpinnings of repeated stimulation remain understudied. In this work, we evaluated the effects of the repetition and intensity of tDCS on cerebral perfusion [cerebral blood flow (CBF)]. A cohort of 47 subjects was randomly assigned to one of the three groups. tDCS of 1- or 2-mA was applied to the left prefrontal cortex on three consecutive days, and resting CBF was quantified before and after stimulation using the arterial spin labeling MRI and then compared with a group that received sham stimulation. A widespread decreased CBF was found in a group receiving sham stimulation across the three post-stimulation measures when compared with baseline. In contrast, only slight decreases were observed in the group receiving 2-mA stimulation in the second and third post-stimulation measurements, but more prominent increased CBF was observed across several brain regions including the locus coeruleus (LC). The LC is an integral region in the production of norepinephrine and the noradrenergic system, and an increased norepinephrine/noradrenergic activity could explain the various behavioral findings from the anodal prefrontal tDCS. A decreased CBF was observed in the 1-mA group across the first two post-stimulation measurements, similar to the sham group. This decreased CBF was apparent in only a few small clusters in the third post-stimulation scan but was accompanied by an increased CBF, indicating that the neural effects of stimulation may persist for at least 24 h and that the repeated stimulation may produce cumulative effects.

## Introduction

Transcranial electrical stimulation (TES) refers to a spectrum of techniques focused on delivering electrical currents non-invasively to the brain with the goal of modulating neural activity. TES has experienced an increased interest over the past 15 years in basic to applied clinical research (Fregni et al., 2015). TES that uses a weak, constant current delivered to the scalp is referred to as the transcranial direct current stimulation (tDCS) (Coffman et al., 2012). The specific application of tDCS with the anode placed on the frontal scalp sites (“anodal prefrontal tDCS”) has been routinely applied in the literature with demonstrable behavioral effects in combatting performance decrements associated with vigilance (Nelson et al., 2014), decreasing the effect of fatigue on the cognitive performance (McIntire et al., 2014, 2017a,b), accelerating learning processes (Bullard et al., 2011; Clark et al., 2012; Coffman et al., 2012; McKinley et al., 2013), enhancing multitasking performance (Nelson et al., 2016), and improving procedural memory (McKinley et al., 2017b).

Despite the broad applications of tDCS and those specific to anodal prefrontal stimulation, the neural mechanisms underlying tDCS are not fully understood. It has been suggested that anodal tDCS increases excitability in the neocortex (Liebetanz, 2002) by altering neuronal membrane potentials (Bindman et al., 1962). This theory is supported by the evidence of enhanced glutamatergic activity, measured from proton magnetic resonance spectroscopy, following the application of anodal tDCS at rest (Clark et al., 2011). Synaptic plasticity, the ability of the brain to form and restructure synaptic connections (Pittenger and Duman, 2008), is thought to coincide with the increased glutamatergic activity (Hunter et al., 2013) and, thus, is theorized as a mechanism of action in tDCS, as evident in the lasting behavioral effects (e.g., McIntire et al., 2014, 2017b) and the acceleration of learning processes (Bullard et al., 2011; Clark et al., 2012; McKinley et al., 2013). Despite the expansive literature on the anodal prefrontal stimulation and neural mechanisms of tDCS, studies exploring the behavioral effects and neural underpinnings of multiple sessions of stimulation are limited. The goal of the present study was to further our understanding of the neural effects of repetitive stimulation to evaluate the potential dosage and tolerance effects.

### Non-invasive Measurement of Cerebral Perfusion

The measurement of cerebral perfusion [volume of blood delivered to a volume of tissue per unit time, referred to as the cerebral blood flow (CBF)] is a growing method for studying neural processes. O^15^-H_2_O PET is the standard for quantifying CBF; however, this imaging requires the injection of a radioactive tracer. Alternatively, the *in vivo* quantification of CBF can be performed non-invasively using MRI through an arterial spin labeling (ASL) pulse sequence (Weber et al., 2013; Grade et al., 2015). ASL uses a simple modification to the standard MRI acquisitions to turn the blood in the neck into an MR tracer. This is completed by labeling blood in a slab inferior to the imaging field of view using magnetic inversion. This inversion will decrease the measured signal, and, thus, CBF can be extracted by comparing the labeled image with a control (unlabeled) image. ASL imaging is clinically used to identify the early pathophysiological changes in Alzheimer's disease (Du et al., 2006; Noguchi et al., 2008; Xu et al., 2010) and other disorders such as dementia (Xu et al., 2010; Borogovac and Asllani, 2012; Weber et al., 2013). In comparison to signals based on blood oxygen, CBF has better reliability and intersubject variability (Weber et al., 2013). Furthermore, CBF is directly responsible for the delivery of glucose and oxygen. Both oxygen and glucose are necessary to maintain ATP production and needs to be replenished to support the continued neural activity. Although CBF is not a direct measure of neural activity, it is a tightly coupled correlate: CBF changes with neural activity which occurs with a changing metabolism (i.e., resting activity) or during task activation (Borogovac and Asllani, 2012).

### Rationale and Hypothesis

Behaviorally, our group has observed various effects from anodal prefrontal tDCS. Increased information throughput (Nelson et al., 2019) and multitasking throughput capacity (Nelson et al., 2016) during the multi-attribute task battery were observed in groups receiving 2-mA compared with those of sham stimulation. Improvements in the target detection were observed during an air traffic controller task in subjects receiving 2-mA compared to those in sham stimulation (Nelson et al., 2014). A similar improvement in the target detection was observed in a vigilance task from a group receiving a 2-mA stimulation compared to those receiving a lower-amplitude stimulation (0.5, 1, and 1.5 mA) as well as sham stimulation (McKinley et al., 2017a). McIntire et al. (2017a,b) observed attentional decrements due to sleep deprivation stress in a sham stimulation group; however, 6 h of improved attentional accuracy and reaction time following a single application of 2-mA stimulation was reported. In addition, self-reports from the 2-mA group revealed more vigor, less fatigue, and reduced boredom than those from the sham group. These effects were found to be reliable and repeated in a duplicated study on a new subject sample (McIntire et al., 2019). A final study observed a decreased sleep time without negative effects on mood or sleep quality following a single session of a 2-mA stimulation compared to the sham group (McIntire et al., 2020).

The study of the resting CBF in anodal prefrontal tDCS may provide critical, novel insights that could help elucidate the mechanisms of the anodal prefrontal tDCS resulting in these various behavioral effects. For instance, increased neural activity associated with anodal tDCS would increase the resting metabolism and, thus, would enhance CBF (Gsell et al., 2000; Nielsen and Lauritzen, 2001; Sheth et al., 2004). Few previous studies have used ASL to assess such neural effects of tDCS. In one study, increased regional CBF within and between subjects was found underneath the site of anodal tDCS, with transfer effects observed in brain regions functionally connected to the stimulation site following a single stimulation of 0.8- to 2-mA (Zheng et al., 2011). In another study where 1-mA anodal and cathodal stimulations were provided to the prefrontal cortex 1 week apart, immediate and lasting changes in CBF were found to be associated with the anodal left prefrontal tDCS (Stagg et al., 2013). Despite observing increased CBF in regions anatomically close to the dorsolateral prefrontal cortex, a widespread CBF was observed after both anodal and cathodal tDCS. Through the comparison of multiple levels of stimulation across concurrent days with that of the sham stimulation, we sought to identify tolerance or cumulative effects of tDCS on the resting CBF.

## Materials and Methods

### Participants

The previous research from our group, in between-subject experiments, has used Cohen's *d* in power analysis to help determine the sample size. Cohen's *d* of 0.8 or larger is considered a large effect. Using a two-sample *t*-test with a power of 0.8, an alpha error of 0.05, and a Cohen's *d* value of 0.8 results in 26 subjects per group. In the current study, there are three groups. Due to constraints of time and funding, and a plan to run follow-up studies, it was decided to use 20 subjects per group.

This study reports the findings from 47 healthy volunteers (mean age = 27.9 ± 4.85, 9 women). In total, we recruited 77 healthy, active-duty, Air Force military members aged 18–42 that did not meet our exclusion criteria (see Supplementary Table 1 for a full list of exclusion criteria). Participants were recruited from Wright Patterson Air Force Base, Ohio, and were randomly assigned to one of our three experimental groups. Withdrawals and disqualifications (detailed below) during the experimental progress resulted in exceeding our planned recruitment of 60 subjects. Additional disqualifications were made during our data analysis due to data issues, resulting in 47 participants being included in this report.

Written informed consent was obtained from each participant prior to any experimental procedures. At the time of consent (~1–2 days prior to the first experimental session), participants were randomly assigned to one of the three groups, received written instructions, and practiced tasks including 5-min of training on the Mackworth Clock test (McIntire et al., 2017b; McKinley, 2018). The experimental protocol was approved by the Air Force Research Laboratory Institutional Review Board at Wright-Patterson Air Force Base under Protocol # FWR20130126H. Participants eligible for compensation (i.e., if participation occurred in an off-duty status) received equal remuneration.

Of the 77 participants recruited and consented, the reported cohort was reduced due to medical disqualification (*n* = 1), withdrawal prior to MRI procedures (*n* = 6; e.g., family issues, being uncomfortable with MRI procedures once seen in person, or illness), self-withdrawal due to illness/family issues or being uncomfortable with MRI procedures (e.g., noise; *n* = 6), incomplete data collection due to MRI scheduling conflicts (*n* = 1), or being medically disqualified due to incidental findings during the initial MRI scan (*n* = 1). Additionally, participants were removed due to missing or corrupted data (*n* = 13) and bad registration between ASL and anatomical images (*n* = 2). Data from the remaining 47 participants were evaluated.

### Experimental Design

**Figure 1:**
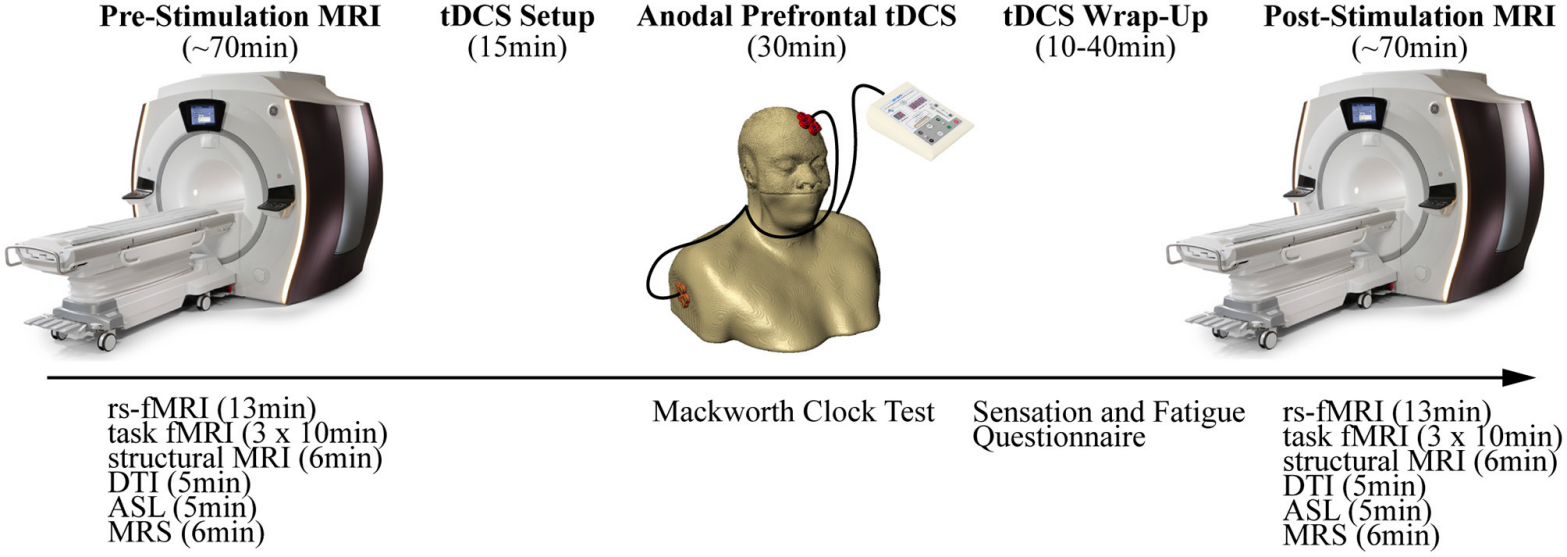
Overview of the experimental design depicting the procedures. Each session began with a pre-stimulation MRI. Participants were then removed from the MRI and anodal prefrontal transcranial direct current stimulation (tDCS) was applied. Finally, a post-stimulation MRI repeated the procedures from the pre-stimulation MRI.

This study was a parallel-group sham-controlled design with two active tDCS conditions (1- and 2-mA for 30 min) and one sham condition (30 s of 2-mA followed by 29.5 min of no stimulation). Each participant completed three experimental sessions, with each session separated by ~24 h. The procedures at each session were identical – first, an initial MRI was performed followed by tDCS executed outside of the MRI, and finally, a second MRI with identical procedures similar to the first MRI (see Figure 1).

The sessions were conducted in the evenings to not only reduce the work-related conflicts but also conform to the availability of MRI. For most of the sessions, two participants were grouped on the same days with staggered start times (see Table 1). We attempted to hold start times consistent within the participants across the three sessions; however, the variability in MRI availability and participant delays could not be fully accounted.

**Table 1:** Starting times for the experimental procedures.

	**Procedure**	**Start time**	**ASL scan time**	**End time**
Participant 1	Pre-stimulation MRI	5:00 p.m.	6:10 p.m.	6:15 p.m.
	Transcranial DC stimulation	6:30 p.m.	n/a	7:00 p.m.
	Post-stimulation MRI	7:30 p.m.	8:40 p.m.	8:45 p.m.
Participant 2	Pre-stimulation MRI	6:15 p.m.	7:25 p.m.	7:30 p.m.
	Transcranial DC stimulation	7:45 p.m.	n/a	8:15 p.m.
	Post-stimulation MRI	8:45 p.m.	9:55 p.m.	10:00 p.m.

*Participants completed the three sessions in groups of two with staggered start times*.

Each of the three groups received the same instructions and performed the same tasks with the exception of stimulation. In the two experimental groups, 1-mA (ACT_1mA_, *n* = 15, mean age = 26.93 ± 3.53, 2 women) or 2-mA (ACT_2mA_, *n* = 17, mean age = 28.61 ± 5.79, 4 women), stimulation was provided for 30 min, while in the control group (CON, *n* = 15, mean age = 28.14 ± 5.08, 3 women), sham stimulation consisting of 2-mA was applied for 30 s followed by 29.5 min of no stimulation. The study was a single-blinded study – the participants, not the experimenters, were uninformed of the validity and intensity of the simulation. Since handedness was not controlled, self-reported handedness was queried. The CON group consisted of one left-handed participant, ACT_1mA_ had none, and ACT_2mA_ had seven.

#### Transcranial Direct Current Stimulation

On each of the three sessions, anodal stimulation was applied to the left prefrontal cortex (approximately F3) in a monopolar montage (i.e., extracephalic cathode). The DC stimulation (MagStim DC Stimulator, Magstim Company Limited, Whitland, UK) was delivered in a manner consistent with the previous reports (McIntire et al., 2017b, 2019; McKinley et al., 2017a; Sherwood et al., 2018). A constant current of 1- or 2-mA depending on the assigned condition was supplied through a ring of five custom Na/NaCl electrodes (Rio Grande Neurosciences, Inc., Sante Fe, NM). The electrodes were arranged in a 1.6-cm radius circle and separated by 0.1 cm (outer edge to outer edge), and the stimulation was distributed evenly among the five anode electrodes (see Petree et al., 2011 for further details on electrodes). Multistage current monitoring is used to ensure that constant current levels are delivered to the anode. The same ring configuration was used at the cathode location, which was placed on the contralateral upper bicep. The extracephalic reference was used to exclude any effects that may be due to the reference (i.e., cathode) electrodes (Nitsche et al., 2007; Priori et al., 2008). Each electrode was placed in a small plastic “cup” and secured to the participant using medical bandages. The electrode cups were filled with a highly conductive gel (SignaGel, Parker Laboratories, Fairfield, NJ) to ensure the current transfer to the scalp and bicep, and air bubbles were removed using a small wood dowel. Sham stimulation lasted 30 s and followed the same procedures but consisted of a 15-s ramp up to a 2-mA current and a 15-s ramp down to mimic the skin sensations during the active stimulation conditions that are due to the current ramp-up. During stimulation, the participants completed a 30-min laboratory vigilance task (Mackworth, 1948).

#### MRI Acquisition

MRI data were acquired at each session prior to and ~30 min following the application of tDCS on a 3 Tesla (T) MRI (Discovery 750 W, GE Healthcare, Madison, WI) equipped with a 24-channel head coil. The MRI acquisition consisted of the following sequences: a 12-min resting-state functional MRI (fMRI) (Kim et al., 2021), three 10-min task fMRIs (Sherwood et al., 2018), T1-weighted MRI (6.5 min for session 1 pre-stimulation, 3.5 min for the remaining sessions), diffusion tensor imaging (DTI; 5 min), single-voxel magnetic resonance spectroscopy (MRS; 6 min), and resting ASL (5 min). As this work is part of a much larger study, we will only be presenting the resting ASL data herein.

Images of CBF were acquired ~20 min prior to the application of tDCS and ~1.5 h after the conclusion of stimulation using a pseudo-continuous arterial spin labeling (pcASL) technique (Silva and Kim, 1999). This sequence administers inversion (tagging) pulses immediately inferior to the imaging volume. All images were acquired with a true axial orientation (i.e., perpendicular to the scanner bore) using a post-label delay time (PLD) of 2,025 ms. Five background suppression pulses were applied to reduce the signal of stationary tissues (Dixon et al., 1991; Mani et al., 1997; Ye et al., 2000) and improve the signal-to-noise ratio (SNR) of arterial blood. A 3D fast spin echo (3D FSE) sequence was used for the acquisition of the imaging volume. To reduce motion sensitivity, to improve acquisition time, and to minimize susceptibility artifacts, a stack-of-spiral readout gradient starting at the center of k-space was used (Glover, 2012). A total of eight spiral arms were used for the k-space sampling. Echoes were re-binned to the Cartesian space in a 128 × 128 matrix, with TR = 4,640 ms, TE = 10.7 ms, voxel size = 1.875 × 1.875 mm^2^, slice thickness = 4 mm, and flip angle = 111°. The sequence acquired a total of 3 tag/control pairs. The total acquisition time was 4 min 46 s. During the scan, the participants were instructed to remain awake and focus on a fixation dot presented on the display. This condition has demonstrated a significantly greater reliability in the resting-state functional MRI across all within-network connections, as well as within default-mode, attention, and auditory networks when compared to eyes open (no specified fixation) and closed methods (Patriat et al., 2013).

Structural (T1-weighted) images were acquired using a 3D brain volume imaging (BRAVO) pulse sequence, which uses an inversion recovery prepared fast spoiled gradient-echo (FSPGR). The structural images were acquired using a 256 × 256 element matrix, 172 slices oriented to the anterior commissure (AC)–posterior commissure (PC) plane, 1 mm^3^ isotropic voxels, 0.8 phase field of view factor, an inversion time (TI) of 450 ms, a TE of 3.224 ms, a flip angle of 13°, and an autocalibrated reconstruction for Cartesian sampling with a phase acceleration factor of 1.0 for the session 1 pre-stimulation session and 2.0 for all the remaining sessions. The longer scan (lower acceleration factor) was used to acquire one higher quality image for other portions of the project. The acceleration factor was increased for the remaining sessions to produce images that are of high enough quality for registration purposes but also to reduce the scan time as much as possible.

### Data Processing and Analysis

Cerebral blood flow maps (see Supplementary Figure 1) were computed and quantified from the automated functions in the GE reconstruction software. First, the three tagged and three control volumes were averaged in place (without motion correction). Then, difference images were calculated for all participants by subtracting the average tagged volume from the average control volume. Finally, quantitative CBF maps (see Supplementary Figure 1 for example of raw CBF maps) were generated from the difference images, the associated proton density (PD)-weighted volumes, and the standard single-compartment model (Alsop and Detre, 1996; Mutsaerts et al., 2014; Alsop et al., 2015) using the formula:

$$\begin{array}{l} CBF\;=\;6000\;*\;\lambda \frac{\left(1-e^{-\frac{ST\left(s\right)}{T_{1t}\left(s\right)}}\right)e^{\frac{PLD\left(s\right)}{T_{1b}\left(s\right)}}}{2T_{1b}\left(s\right)\left(1-e^{-\frac{LT\left(s\right)}{T_{1b}\left(s\right)}}\right)\;\varepsilon \;*\;{NEX}_{PW}}\left(\frac{PW}{{SF}_{PW}PD}\right) \end{array}$$

where CBF is calculated in ml/100g/min. In this equation, *T*_1*b*_ is the T1 of blood and is assumed to be 1.6 s at 3 T. The partial saturation of the reference image (PD) is corrected using a *T*_1*t*_ of 1.2 s (typical of gray matter). The saturation time, *ST*, is set to 2 s, and the partition coefficient λ is set to a whole brain average of 0.9. The efficiency, ε, is the overall efficiency (0.6), a combination of both inversion efficiency (0.8) and background suppression efficiency (0.75). The PLD used for the ASL protocol was 2,025 ms, and the labeling duration, *LT*, was set to 1.5 s in the current version. *PW* is the perfusion weighted or the raw difference, and *SF*_*PW*_ is the scaling factor of the PW sequence. The number of excitations for PW images, *NEX*_*PW*_, was set to 3.

The CBF maps from each session were exported from the MRI scanner and processed using the FMRIB Software Library (FSL; Smith et al., 2004; Woolrich et al., 2009) on a 128-core Rocks Cluster Distribution (www.rocksclusters.org) high-performance computing system capable of running 256 threads in parallel. Then, the high-resolution structural image of an individual was registered to the MNI-152 T1-weighted 2 mm template provided in FSL (Collins et al., 1995; Mazziotta et al., 2001) using a 12-parameter model (Jenkinson and Smith, 2001; Jenkinson et al., 2002; see Supplementary Figure 2A). Next, the raw PW images were registered to the high-resolution structural image by estimating the motion from a boundary-based registration method, which includes a field-map-based distortion correction (Greve and Fischl, 2009) (see Supplementary Figure 2B). In order to co-register all volumes, the CBF maps were converted to the standard space using the transforms responsible for morphing the PD-weighted image of each data set to the structural image and the structural image to the template (see Supplementary Figure 2C).

Voxelwise non-parametric analyses were performed using the conditional Monte Carlo permutation testing based on the method of Freeman and Lane (1983) implemented in randomise of FSL (Anderson and Robinson, 2001; Winkler et al., 2014). Due to the mixed effect of our design and how the data would need to be permuted, we were not able to perform proper between-group repeated measures ANOVAs. However, we implemented the following steps to evaluate run x group interaction effects. First, we subtracted each post-stimulation CBF map (in the standard space) from the corresponding baseline (session 1 pre-stimulation). Next, unpaired *t-*tests were performed to evaluate the between-group differences in the change from baseline per post-stimulation measurement (sessions 1–3 post-stimulation). These tests were conducted to compare ACT_1mA_ and ACT_2mA_ groups with the CON group separately. This resulted in a total of six analyses, analogous to the *post-hoc* pairwise testing that would be conducted to interpret a significant interaction effect. Null *t* distributions for the contrast representative of the between-group difference were derived by performing 2,000,000 random permutations of the data. Each permutation was created by exchanging the assigned group (Nichols and Holmes, 2003). A final *t* statistic was computed for each voxel by testing the unshuffled, real arrangement against the permutation distribution.

Additionally, voxelwise non-parametric, within-group one-way ANOVAs were performed on the session 1 pre-stimulation, session 1 post-stimulation, session 2 post-stimulation, and session 3 post-stimulation co-registered resting CBF maps. This analysis was also conducted using randomise of FSL. Null distributions for contrasts representative of the main effect of session (sessions 1–3 post-stimulation subtracted from the baseline) were derived by performing 2,000,000 random permutations of the data. Each permutation was created by exchanging the assigned session while maintaining subject membership. Then, an *F* test compared the means from each session (sessions 1–3 post-stimulation subtracted from the baseline). Pairwise comparisons were executed during the completion of the one-way ANOVAs. The results of the pairwise comparisons were further corrected for multiple comparisons to account for false positives due to the multiple comparisons (Worsley, 2001). This method considered adjacent voxels with a *t* statistic of 2.3 or greater to be a cluster. The significance of each cluster was estimated and compared to a threshold of *p* < 0.05 using the Gaussian random field theory-based maximum height thresholding and a one-tailed *t-*test. The significance of voxels that either did not pass the significance level threshold or do not belong to a cluster was set to zero.

## Results

### Scanning Time

**Figure 2:**
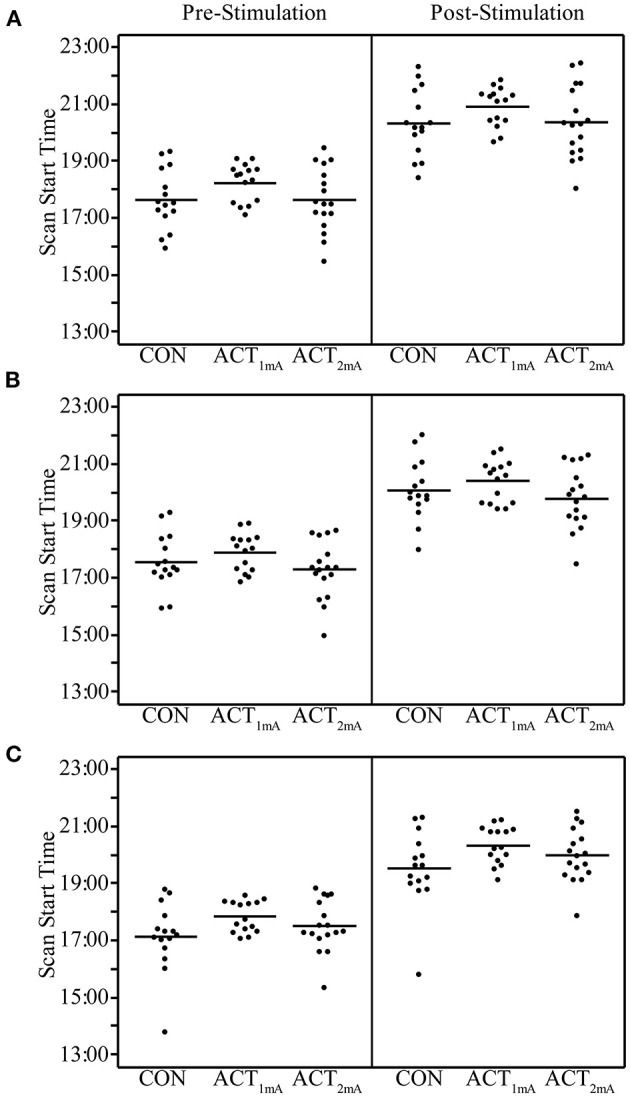
Pre- and post-stimulation scan start times for session 1 **(A)**, session 2 **(B)**, and session 3 **(C)** separated by group. Group averages are indicated by the bar.

**Figure 3:**
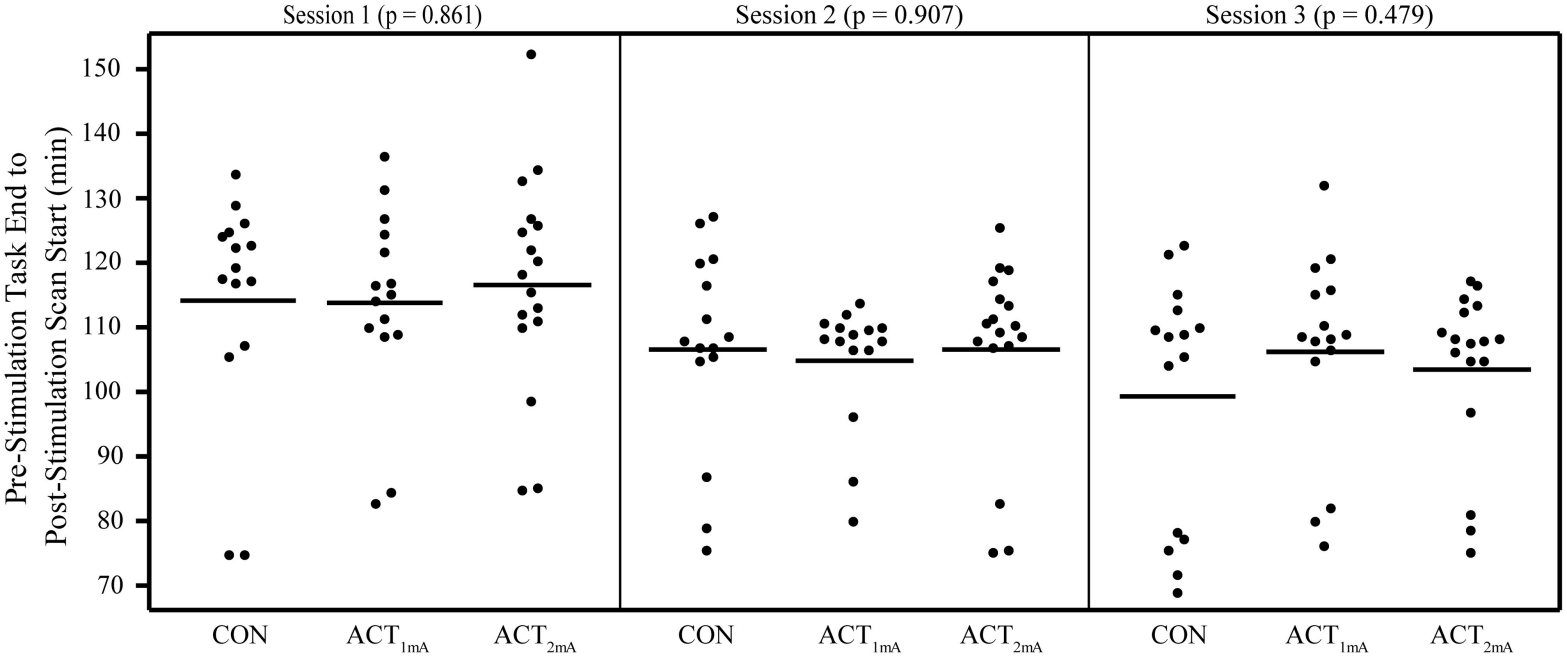
Duration from pre-stimulation task end time to post-stimulation scan start time separated by groups and sessions. No significant differences were revealed in one-way ANOVAs comparing groups per session.

Concerns regarding the potential bias in the data due to the within-subject variability in scanning start time arose during the analysis (see Supplementary Table 2 for a complete list of the scan initiation times). To address this concern and the potential impact of the session time on any one group in particular, we evaluated the scan initiation times between groups by the session and scan. First, we extracted the scan initiation times from the log files. Next, we performed between-group one-way ANOVAs separately for the pre- and post-stimulation scan initiation times for each session. We did not correct for the family-wise error to be more sensitive to timing differences between groups. Our analyses did not find any significant variability (*p* > 0.05; Table 2) between groups for the scan start time for any of the scan sessions (Figure 2). There was also concern raised regarding the variable durations between the stimulation and the post-stimulation ASL that may disproportionally affect the results. Unfortunately, the stimulation time was not recorded in reference to the scan time. Therefore, we used the end time for the pre-stimulation task scan as the best approximation since the setup time for tDCS and post-task scanning was fairly consistent and much less variable than the total scan times. The duration between the pre-stimulation task end time and the post-stimulation scan start time was computed, and one-way ANOVAs were conducted to compare the groups across each session. Again, we did not correct for a family-wise error. The results did not reveal any significant variability (*p* > 0.05) in duration between the pre- and post-stimulation scans (Figure 3).

**Table 2:** Results of the one-way ANOVAs comparing the initiation time of each scan between groups.

		***p*-value**
Session 1	Pre-stimulation	0.158
	Post-stimulation	0.258
Session 2	Pre-stimulation	0.172
	Post-stimulation	0.231
Session 3	Pre-stimulation	0.132
	Post-stimulation	0.099

*Family-wise error correction was not applied to be more sensitive toward differences in groups*.

### Changes in CBF

**Figure 4:**
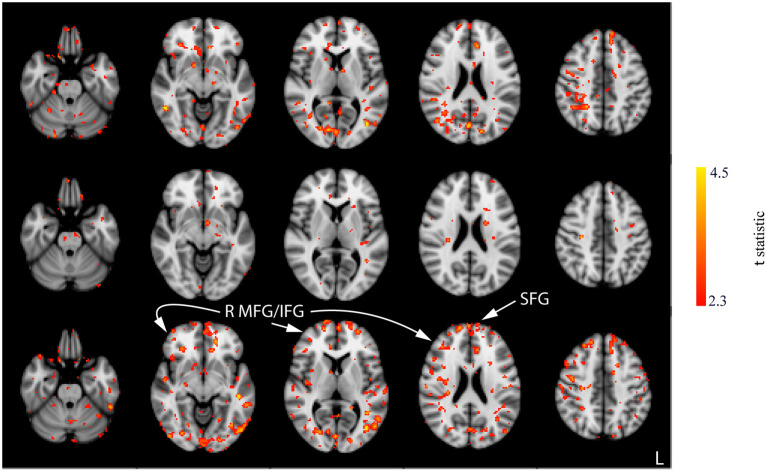
Results of the unpaired *t-*tests between CON and ACT_1mA_ for the change between session 1 pre-stimulation and session 1 post-stimulation (top row), session 2 post-stimulation (middle row), and session 3 post-stimulation (bottom row). Axial slices are taken from MNI coordinates z = −26, −8, 6, 22, and 44 mm (left to right).

**Figure 5:**
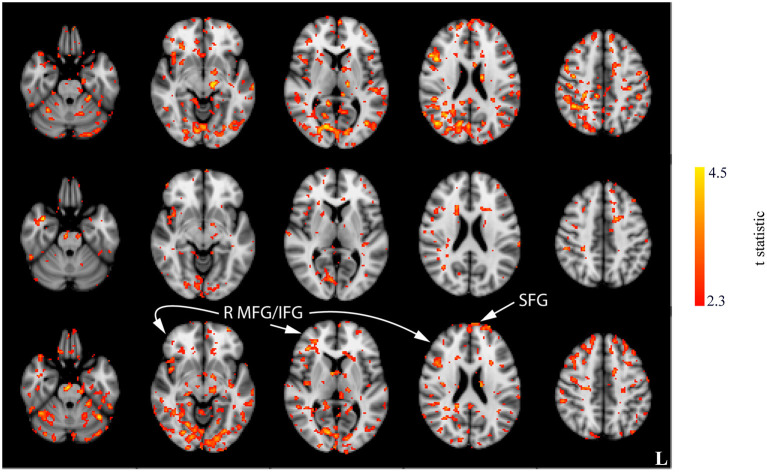
Results of the unpaired *t-*tests between CON and ACT_2mA_ for the change between session 1 pre-stimulation and session 1 post-stimulation (top row), session 2 post-stimulation (middle row), and session 3 post-stimulation (bottom row). Axial slices are taken from MNI coordinates z = −26, −8, 6, 22, and 44 mm (left to right).

Interaction effects between the run and stimulation were assessed *via* the unpaired *t-*tests following a subtraction of the post-stimulation measurement from the pre-stimulation measurement from session 1 (i.e., baseline). The comparison of resting CBF from baseline and post-stimulation between CON and ACT_1mA_ groups resulted in significant variations across the three sessions (Figure 4). A similar trend was observed between CON and ACT_2mA_ (Figure 5). Frontal areas in these analyses appeared with an increasing statistical significance and extent, with stronger effects observed in the comparison between CON and ACT_2mA_. These consisted of the bilateral superior frontal gyrus (SFG) and the right middle frontal and inferior frontal gyri (MFG and IFG, respectively). Posterior regions such as the right superior parietal lobule, the inferior parietal lobule, the middle temporal gyrus, and the precuneus demonstrated a trend with a decreasing statistical significance and extent.

**Figure 6:**
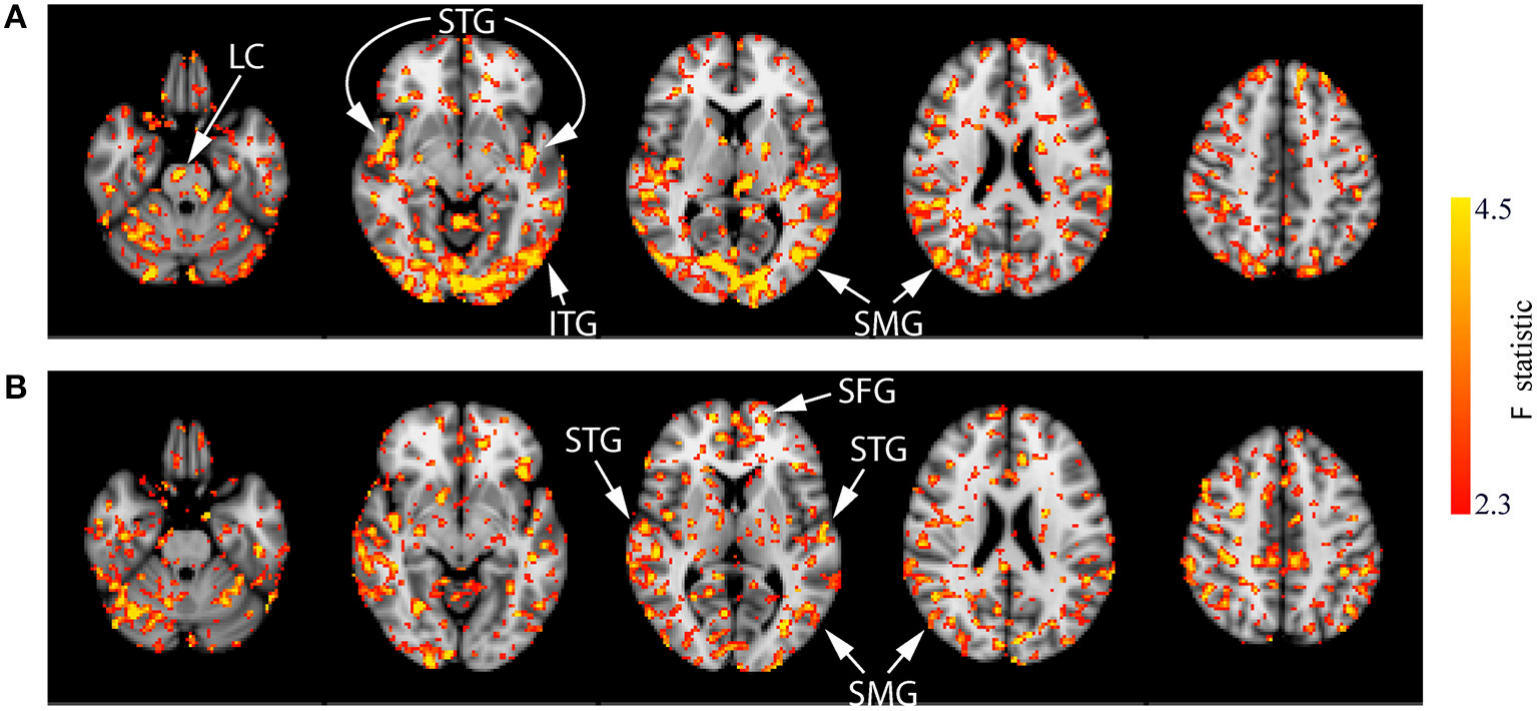
Results of the one-way ANOVA demonstrating the main effect of session for the CON **(A)** and ACT_1mA_ **(B)** groups. Axial slices are taken from MNI coordinates z = −26, −8, 6, 22, and 44 mm (left to right).

These results are difficult to interpret alone and could represent either a greater decrease from baseline or a smaller increase from baseline between the CON group and the active group. Therefore, the within-group analyses were conducted to provide a clearer understanding of these effects. For the CON and ACT_1mA_ groups, repeated-measure one-way ANOVAs revealed significant differences in CBF across four different measurements (baseline and sessions 1–3 post-stimulation; see Figure 6). Regions identified in these analyses include the LC, superior temporal gyrus (STG), inferior temporal gyrus (ITG), supramarginal gyrus (SMG), and SFG. There were no significant findings for the main effect of the session in the ACT_2mA_ group. However, pairwise comparisons were further conducted to evaluate CBF each post-stimulation measure in comparison to the baseline for each group. The results summarized from these analyses identifying a decreased CBF from baseline are shown in Table 3 and the increased CBF from baseline are shown in Table 4.

**Table 3:** Summarized findings highlighting regions with significantly decreased CBF resulting from the pairwise comparisons of each post-stimulation measurement with baseline.

	**Session 1 post-stimulation**	**Session 2 post-stimulation**	**Session 3 post-stimulation**
Sham	Several clusters including ITG, SMG, and pre-central gyrus	Several clusters including STG and LC	Widespread including ITG, SMG, LC, STG, and pre-central gyrus
ACT_1mA_	Few small clusters	Widespread including STG, ITG, and SMG	Few small clusters
ACT_2mA_	n.s.	Clusters in the pre-central gyrus and STG	Very few, small clusters

*No significant findings are reported as n.s*.

**Table 4:** Summarized findings highlighting regions with significantly increased CBF resulting from the pairwise comparisons of each post-stimulation measurement with baseline.

	**Baseline minus session 1 post-stimulation**	**Baseline minus session 2 post-stimulation**	**Baseline minus session 3 post-stimulation**
Sham	Very few, small clusters	Very few, small clusters	Very few, small clusters
ACT_1mA_	Few small clusters	Very few, small clusters	Few clusters including the ACC and SFG
ACT_2mA_	n.s.	Few small clusters	Several clusters including the LC, IFG, insula, SFG, thalamus, hippocampus, and fusiform gyrus

*No significant findings are reported as n.s*.

**Figure 7:**
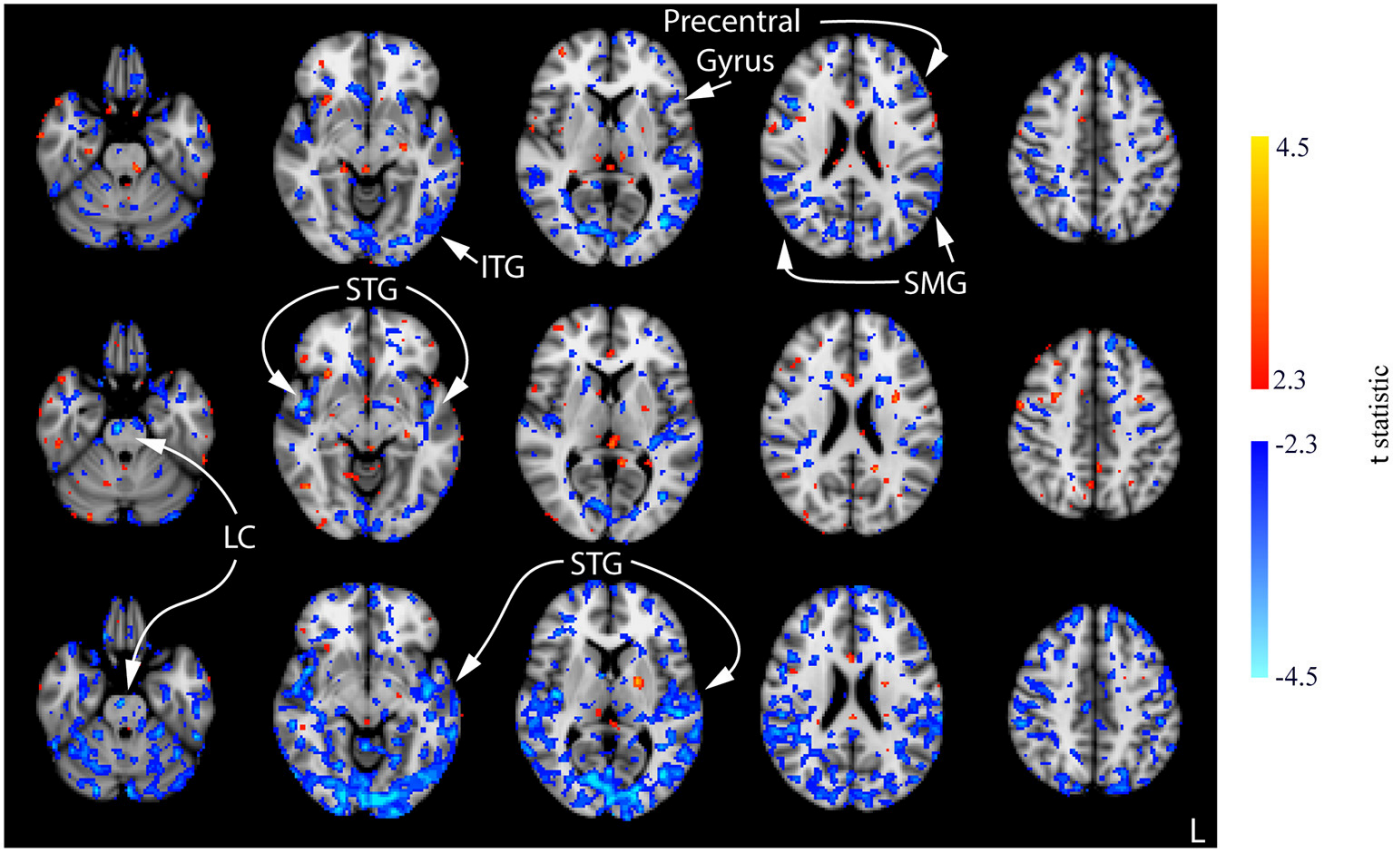
Results of the one-way ANOVA for the CON group displayed through *post-hoc*, pairwise comparisons between session 1 pre-stimulation and session 1 post-stimulation (top row), session 2 post-stimulation (middle row), and session 3 post-stimulation (bottom row). Corresponding images demonstrating the magnitude of CBF changes are given in Supplementary Figure 3. Axial slices are taken from MNI coordinates z = −26, −8, 6, 22, and 44 mm (left to right).

Compared to the baseline, decreases in CBF were observed in all three post-stimulation sessions for the CON group (Figure 7; Supplementary Figure 3). The amount CBF was lowered in comparison to that of session 1 pre-stimulation increased in statistical reliability, extent, and magnitude by session 3 post-stimulation. Of note, decreased CBF was observed consistently in the bilateral STG and pre-central gyrus. Decreases in CBF in the ITG and SMG were observed in the post-stimulation measures for sessions 1 and 3. Additionally, decreased CBF in the LC was observed in the post-stimulation measures for sessions 2 and 3.

**Figure 8:**
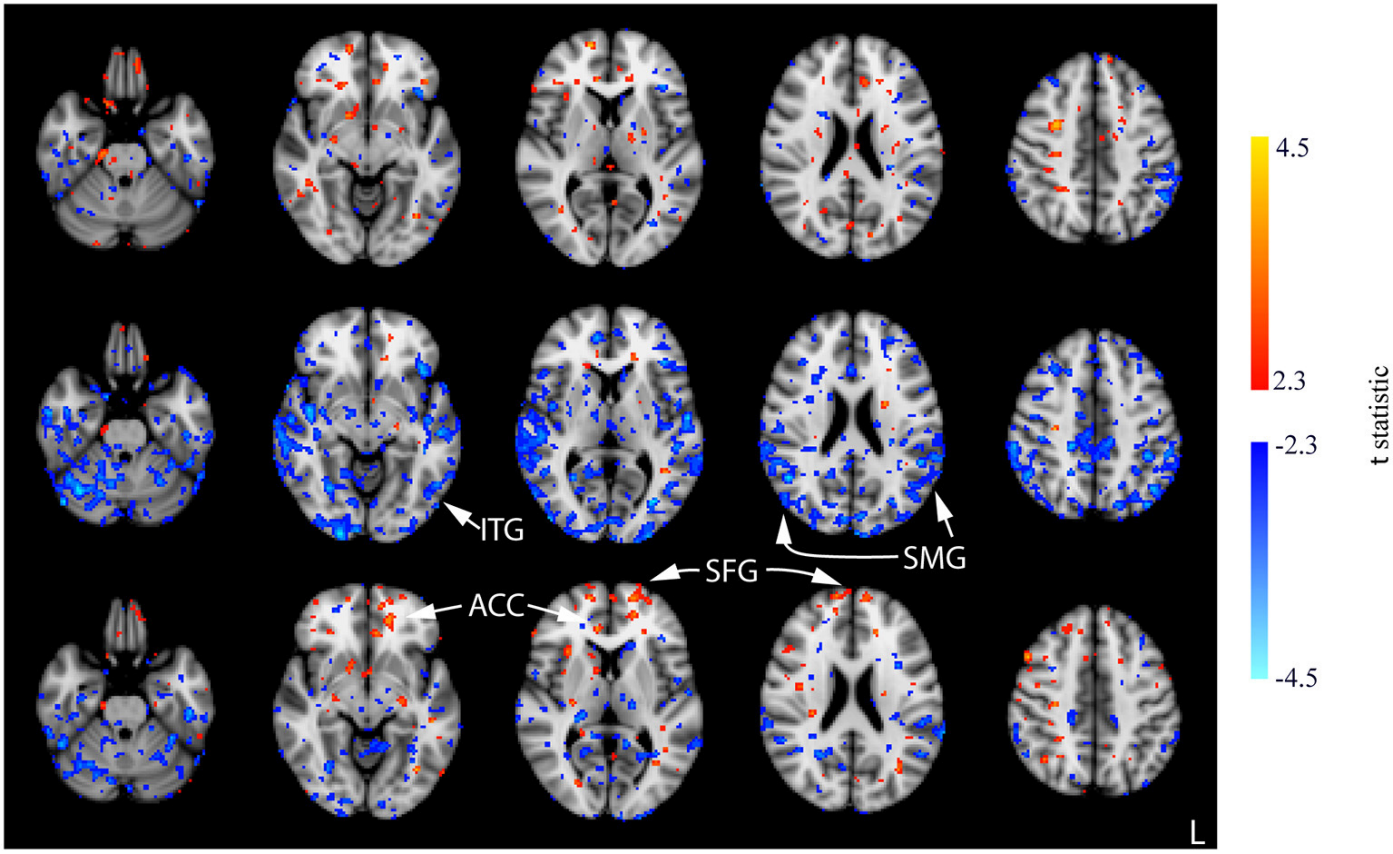
Results of the one-way ANOVA for the ACT_1mA_ group displayed through *post-hoc*, pairwise comparisons between session 1 pre-stimulation and session 1 post-stimulation (top row), session 2 post-stimulation (middle row), and session 3 post-stimulation (bottom row). Corresponding images demonstrating the magnitude of CBF changes are given in Supplementary Figure 4. Axial slices are taken from MNI coordinates z = −26, −8, 6, 22, and 44 mm (left to right).

Few significant findings were present in the ACT_1mA_ group at session 1 post-stimulation but widespread decreases in CBF were observed at session 2 post-stimulation (Figure 8, top and middle rows; Supplementary Figure 4). These decreases share similarities with that observed in post-stimulation measures from sessions 1 and 3 in the sham group including the SMG and ITG. A decreased CBF diminished by session 3 with only a few small clusters remaining (Figure 8, bottom row). The diminished CBF at session 3 was accompanied by an increased CBF appearing bilaterally in the SFG and the anterior cingulate cortex (ACC).

**Figure 9:**
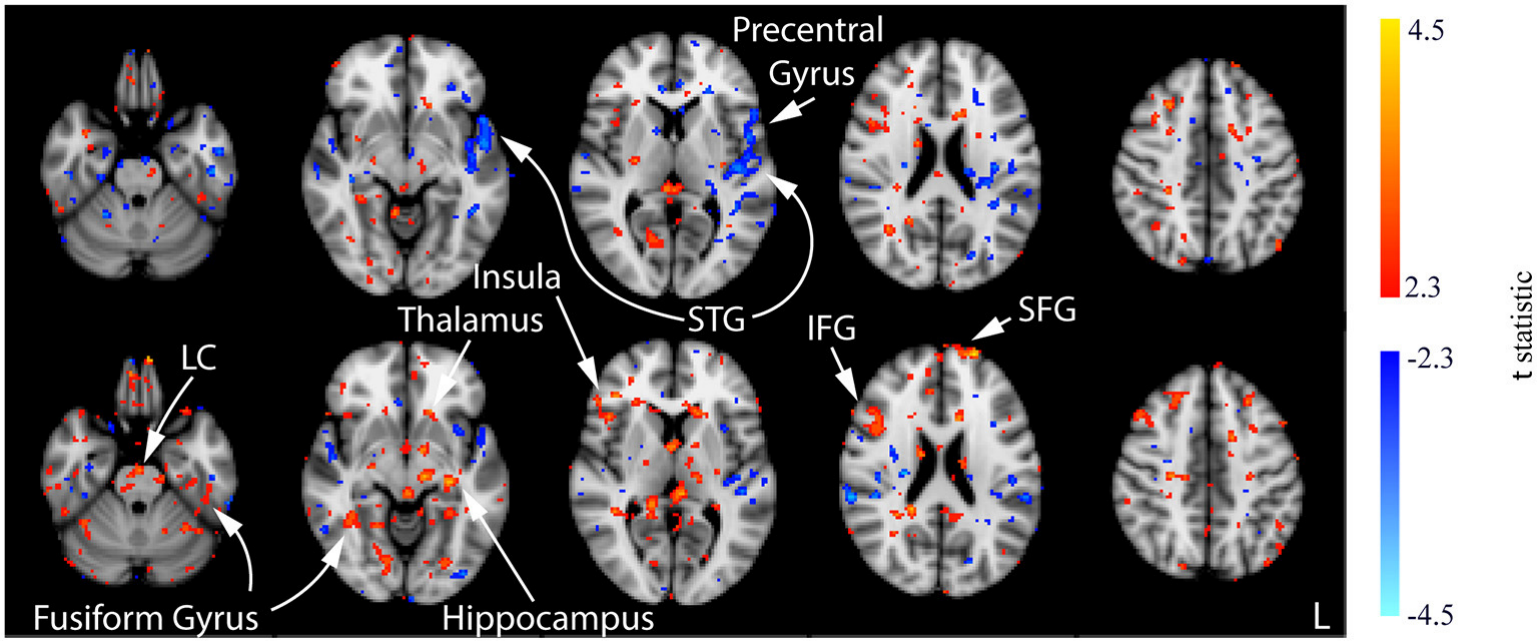
Results of the one-way ANOVA for the ACT_2mA_ group displayed through *post-hoc*, pairwise comparisons between session 1 pre-stimulation and session 2 post-stimulation (top row) and session 3 post-stimulation (bottom row). Corresponding images demonstrating the magnitude of CBF changes are given in Supplementary Figure 5. Axial slices are taken from MNI coordinates z = −26, −8, 6, 22, and 44 mm (left to right).

In contrast with the CON and ACT_1mA_ groups, no significant changes in CBF were observed between baseline and the session 1 post-stimulation measurement for the ACT_2mA_ group. Less defined changes in CBF were observed in the session 2 post-stimulation scan (Figure 9, top row; Supplementary Figure 5). The largest clusters of decreased CBF were observed in the left STG and the left pre-central gyrus; these areas were also observed to have a decreased CBF in the CON group across all three comparisons. For the ACT_2mA_ group, more defined increases were observed by the third post-stimulation session (Figure 9, bottom row). Clusters were observed in the LC, the left hippocampus, the bilateral fusiform gyrus, the left thalamus, the right insula, the right IFG, and the left SFG.

## Discussion

This study examined the effect of tDCS on the resting CBF, quantified using 3D pcASL, at different stimulation intensity levels across three consecutive days. There was significant variability in the resting CBF from baseline (session 1 pre-stimulation) and post-stimulation measures between our sham group and both experimental groups receiving 1- or 2-mA anodal tDCS applied to the prefrontal cortex (Figures 4, 5). In the sham group, significant, widespread decreases in CBF were revealed between the baseline scan (session 1 pre-stimulation) and all three post-stimulation scans. The magnitude, extent, and significance of these decreases rose across the sessions (Figure 6). One postulation for this observation is the cognitive demands of our experimental protocol. Decreased CBF has been associated with a sustained mental workload and an increased time on the task (Paus et al., 1997; Coull and Nobre, 1998; Lim et al., 2010). The time-on-task effect on behavioral performance, an effect of the sustained mental workload, is theorized to arise from the consumption of resources that cannot be immediately replenished and not likely an effect of boredom. Evidence supporting this theory has been observed in PET studies, which have identified a decreased regional CBF as time-on-task increased (Paus et al., 1997; Coull and Nobre, 1998). Further support has been found using ASL, where better performance was associated with smaller decreases in CBF from pre- to post-task imaging (Lim et al., 2010).

It is also possible for the demands of home life, work, and completing the experimental protocol resulted in inadvertent effects on sleep, such as mild reductions in sleep time or quality. While this was not directly measured, either *via* actigraphs or sleep questionnaires, it could help explain our observations. Overt sleep restriction has also been associated with altered neural patterns. Poudel et al. (2012) observed a decreased CBF measured from ASL following acute sleep loss (4 h of restricted sleep) in comparison to the “rested” scans in the same subjects. Shenfield et al. (2020) discovered that changes in the alpha and delta power in electroencephalography (EEG) were related to the subjective sleepiness and performance on the psychomotor vigilance task. EEG signals are summed excitatory and inhibitory postsynaptic potentials, which require metabolic energy and have been found to be positively correlated with the cerebral oxygen uptake and blood flow (Ingvar et al., 1976; Kuschinsky, 1993), suggesting that lower CBF would accompany the decreased EEG power. Decreased delta power has been observed during non-REM sleep in groups restricted to 4 h and 6 h of sleep over 14 continuous days, with effects equivalent to 48 h of sleep deprivation (Van Dongen et al., 2003).

On the contrary to the sham group, the decreased CBF for our group receiving a 2-mA prefrontal tDCS was mostly absent across the post-stimulation comparisons with baseline over the three sessions. Decreased CBF was minimal in the 2-mA group but was observed in its largest magnitude, significance, and extent in the session 2 post-stimulation scan, mainly localized to the left STG and the pre-central gyrus. Recent evidence from our group has shown that sleep time on the night following a 2-mA anodal prefrontal stimulation was decreased compared with that of the sham and stimulation over the primary motor cortex without any significant effects on subjective measures of mood or sleep quality (McIntire et al., 2020). This suggests that the anodal prefrontal stimulation may provide more efficient sleep leading to a lower impact of the potential effects from sleep restriction, which would be present in the form of a decreased CBF. Alternatively, an increased CBF was revealed in post-stimulation measures from sessions 2 and 3, increasing in significance, extent, and magnitude from session 2 to 3. This observation may be explained by an increased CBF in the LC in our ACT_2mA_ group (see Figure 9) compared with the decreased CBF in the CON group (see Figure 7). LC cells of the pons are responsible for triggering the production of norepinephrine and are projected *via* the bilateral ascending pathways to target numerous subcortical and cortical regions (Jenkins et al., 2016). This noradrenergic system allows the LC to modulate multiple distant brain regions simultaneously and can exert its effect by binding to receptors on both pre- and postsynaptic cells (Arnsten, 2000). The lack of decreased CBF, which appeared in the sham group potentially due to time-on-task and/or sleep restriction effects, may be a direct result of stimulation or an effect produced by higher arousal states from an increase in the noradrenergic system. However, the altered noradrenergic system between sham and 2-mA anodal prefrontal tDCS may explain the various behavioral findings from our group (Nelson et al., 2014, 2015, 2016, 2019; McIntire et al., 2017b, 2019; McKinley et al., 2017a).

Results from our 1-mA stimulation group were intriguing. Decreases in CBF were observed at sessions 1 and 2 post-stimulation when compared to baseline, a trend similar to sham and dissimilar to the 2-mA group. These decreases were most prominent by session 2 post-stimulation. However, the magnitude, extent, and statistical significance of these prominent session 2 decreases were lowered at the end of session 3, and a higher CBF was observed in some areas common with the 2-mA stimulation group including the SFG (see Figures 8, 9). This shift in polarity, from decreasing CBF to increasing CBF between sessions 2 and 3, suggests that there may be cumulative effects from tDCS when applied within 24 h. tDCS is believed to modulate the excitability of neural populations by depolarizing neurons below the cathode, increasing the resting membrane potential and neuronal excitability (Nitsche et al., 2008; Nitsche and Paulus, 2011; Brunoni et al., 2012; McKinley et al., 2012; Romero Lauro et al., 2014; Adachi et al., 2015); however, it is currently not known how long these neural changes may persist. Our findings suggest that neural effects of stimulation may persist for at least 24 h allowing consecutive stimulation protocols to compound. This finding adds to previous behavioral findings, which indicated improved arousal appearing for up to 24 h post-stimulation (McIntire et al., 2017a) and improved behavior for at least 6 h post-stimulation (McIntire et al., 2014). More recent evidence suggests that repetitive stimulation may not produce additive benefits (McIntire et al., 2020); however, our findings indicate that stimulation may produce neural effects lasting at least 24 h, and these effects may compound with repeated stimulation.

In conclusion, we observed that the resting CBF decreased from baseline in all three post-stimulation measures from our sham group. In the group receiving 2-mA anodal prefrontal tDCS, little to no decreases in CBF were observed but increases were observed in the post-stimulation measures from sessions 2 and 3. These increases were localized to a few areas. Notably, the LC had a significant increased resting CBF at session 3 post-stimulation compared to baseline, which could indicate increased norepinephrine production and enhanced activity of the noradrenergic system. If found to be true, this could help explain the broad range of behavioral changes observed following anodal prefrontal tDCS. Our group receiving 1-mA tDCS appeared similar to the sham group through session 2 post-stimulation, with observations of decreased resting CBF with little to no increases. By session 3, however, decreases in CBF were minimal and increased resting CBF trends were observed, indicating the potential for the neural effects of tDCS to persist for up to 24 h following stimulation.

## Data Availability Statement

The datasets presented in this article are not readily available because it has not been approved for public release and, therefore, may not be available upon request. The decision to release the data cannot be made by the authors. Requests to access the datasets should be directed to Matthew S. Sherwood, matt.sherwood@us.kbr.com.

## Ethics Statement

The studies involving human participants were reviewed and approved by Air Force Research Laboratory Institutional Review Board, Air Force Research Laboratory, Wright-Patterson Air Force Base. The patients/participants provided their written informed consent to participate in this study.

## Author Contributions

MS, LM, and RM contributed to the design and provided the conception of and overall guidance to the project. MS, LM, and AM contributed to the data collection. MS and AM contributed to the data analysis. MS, LM, AM, KK, CR, and RM contributed to the interpretation of the data. MS contributed to the initial drafting of the manuscript and produced the final artwork. All authors contributed to the writing, revising, approving of the manuscript, and are equally accountable for all aspects of the work.

## Author Disclaimer

The opinions expressed herein belong solely to the authors. They do not represent and should not be interpreted as being those of or endorsed by the Department of Defense or any other branch of the federal government. The U.S. Government is authorized to reproduce and distribute reprints for governmental purposes notwithstanding any copyright notation thereon. The voluntary, fully informed consent of the subjects used in this research was obtained as required by 32 CFR 210 and DODI 3216.02_AFI 40-402.

## Conflict of Interest

MS is employed by the company KBR Inc. MS serves in an unpaid role as a member of Aaron Madaris's Dissertation Committee at Wright State University. AM received compensation for this work as an intern through Infoscitex, Inc. and is also a student at Wright State University. LM is employed by Infoscitex, Inc. The remaining authors declare that the research was conducted in the absence of any commercial or financial relationships that could be construed as a potential conflict of interest.

## Publisher's Note

All claims expressed in this article are solely those of the authors and do not necessarily represent those of their affiliated organizations, or those of the publisher, the editors and the reviewers. Any product that may be evaluated in this article, or claim that may be made by its manufacturer, is not guaranteed or endorsed by the publisher.
