# Supplementary material for: Intensity-Dependent Changes in Quantified Resting Cerebral Perfusion With Multiple Sessions of Transcranial DC Stimulation
Source: Front Hum Neurosci. 2021 Aug 12;15:679977. doi: 10.3389/fnhum.2021.679977 (PMC8397582; doi:10.3389/fnhum.2021.679977)
Supplement: Supplementary file 1 [file Data_Sheet_1.docx]

Supplementary Material


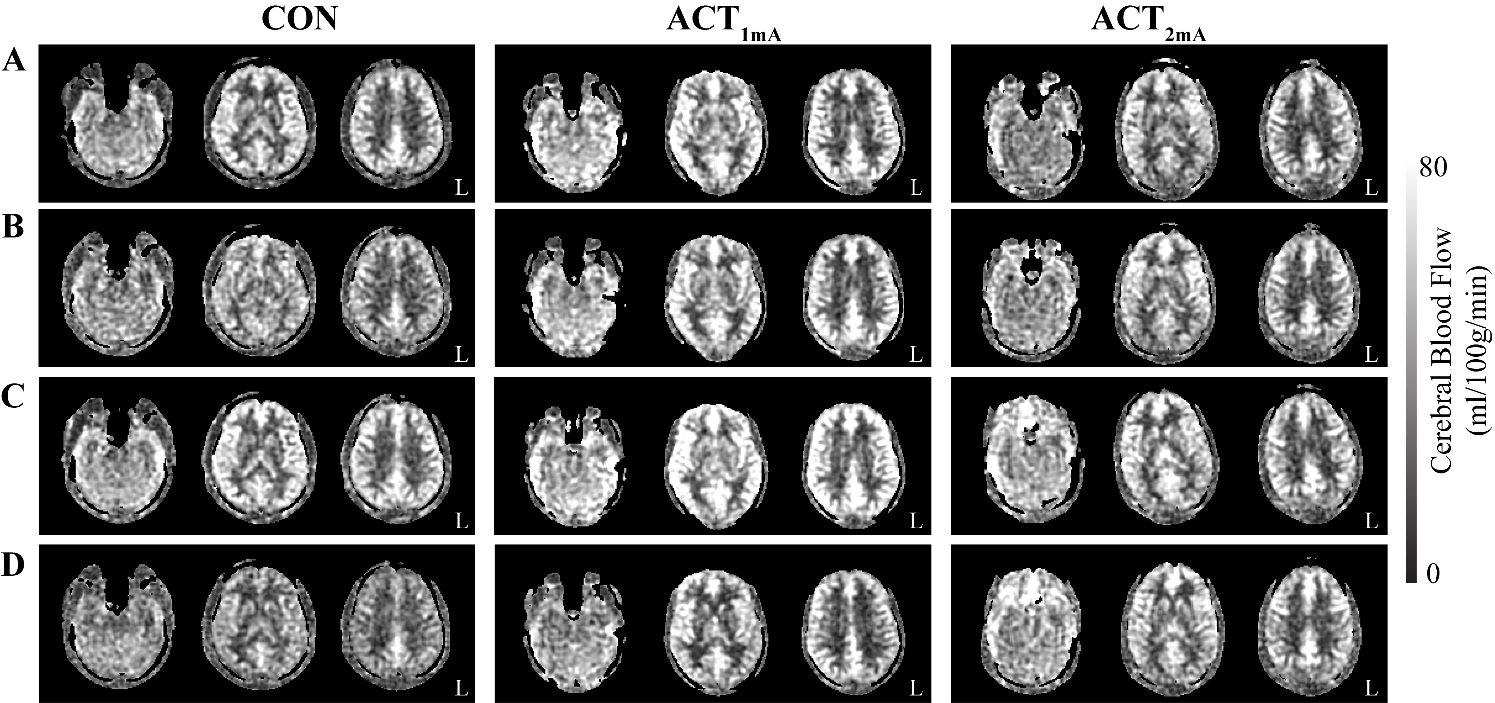


Supplemental Figure 1. Raw CBF maps for a single subject from the CON (left column), ACT_1mA_ (center column), and ACT_2mA_ (right column groups for session 1 pre-stimulation (A), session 1 post-stimulation (B), session 2 post-stimulation (C), and session 3 post-stimulation (D).


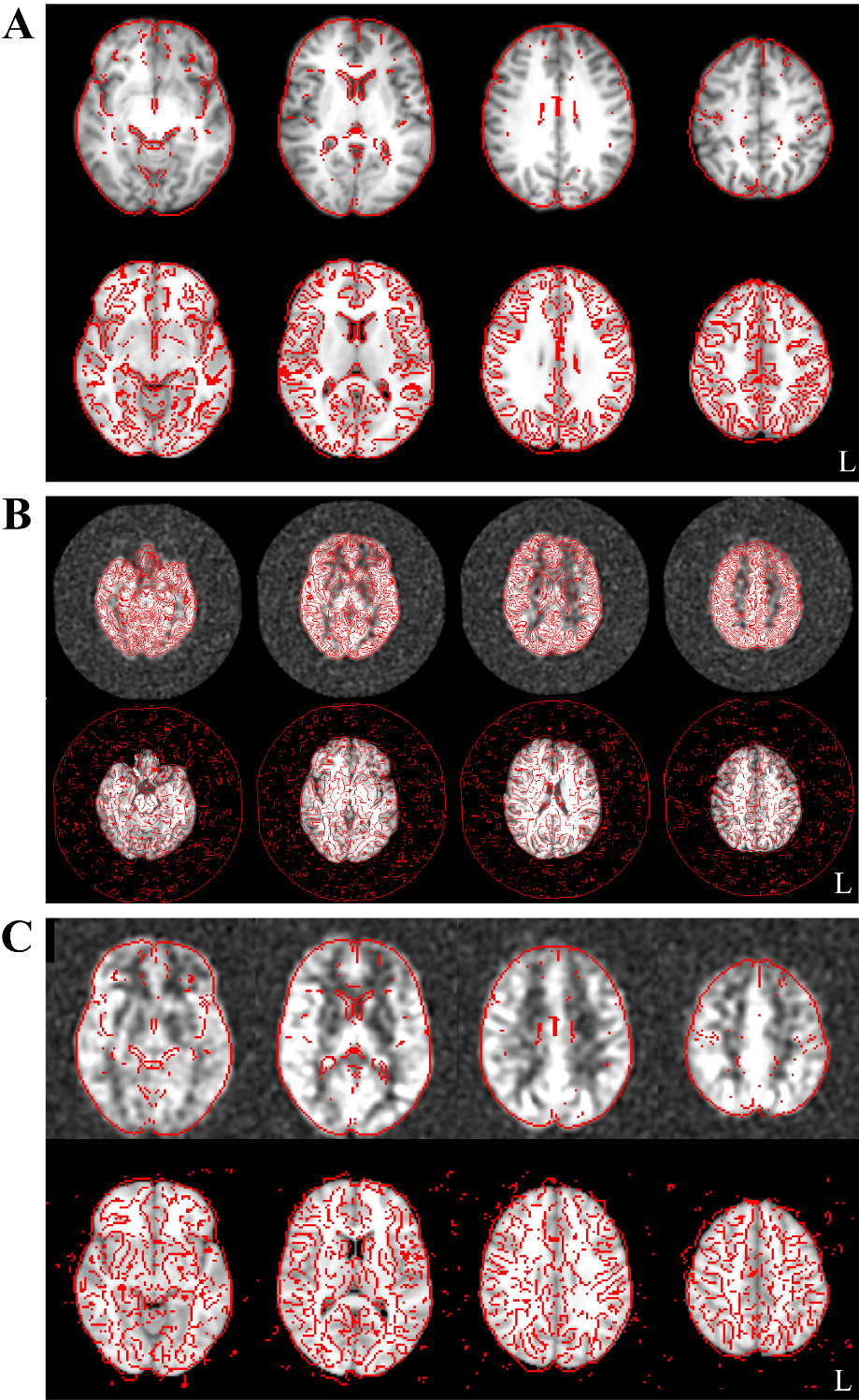


Supplemental Figure 2. Example registration results from one subject (ACT_2_) for structural to standard space (A), ASL to structural (B), and ASL to standard space (C). Top row indicates the input (volume to be transformed) and the bottom row represents the reference volume (target transformed space). Red lines indicate detected boundaries used for registration (top row, reference volume; bottom row, input).


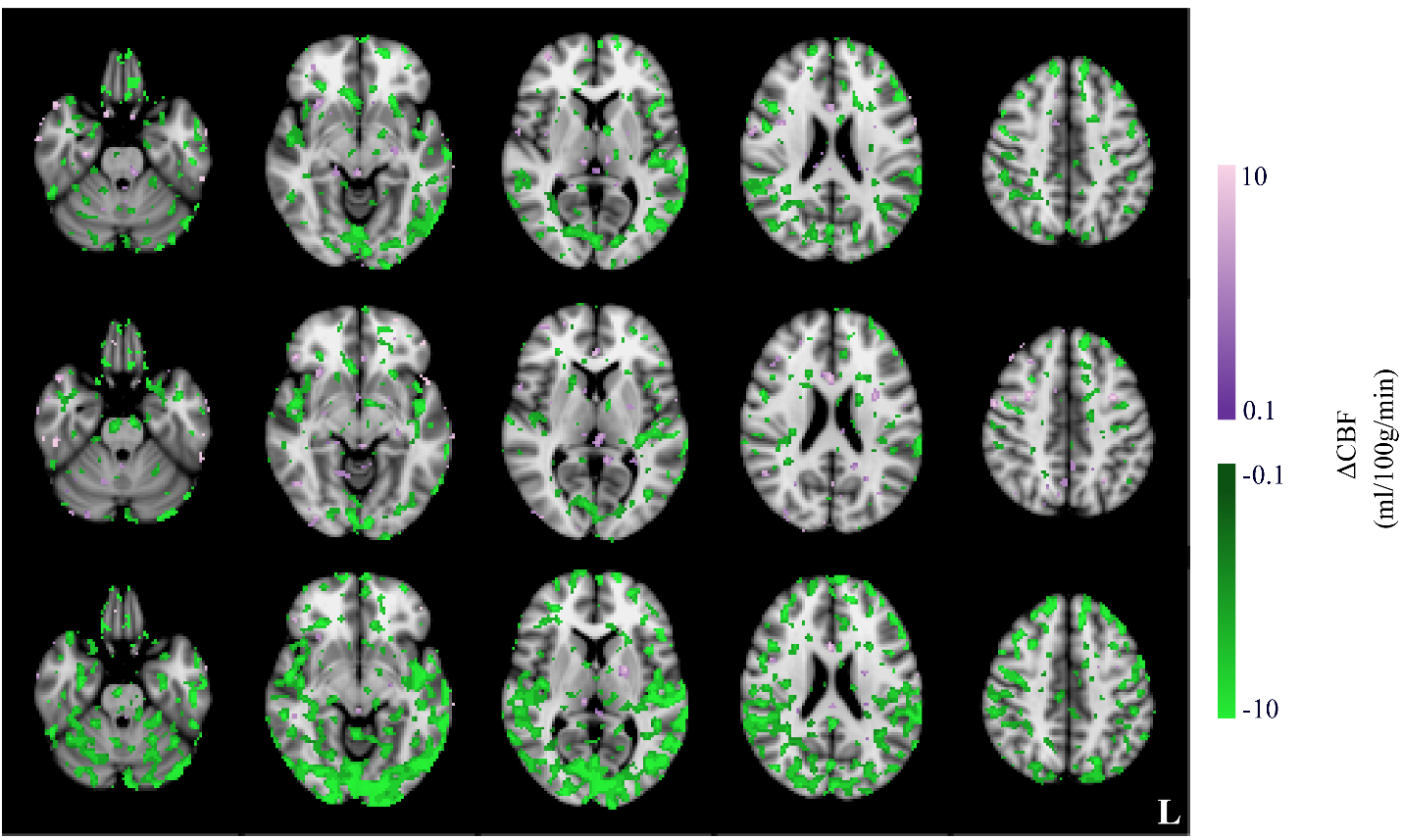


Supplemental Figure 3. CON group-average changes in CBF for significant voxels from the *post hoc,* pairwise comparisons between session 1 pre-stimulation and (top row) session 1 post-stimulation, (middle row) session 2 post-stimulation and (bottom row) session 3 post-stimulation. Axial slices taken from MNI coordinates z = -26, -8, 6, 22, and 44mm (left to right).


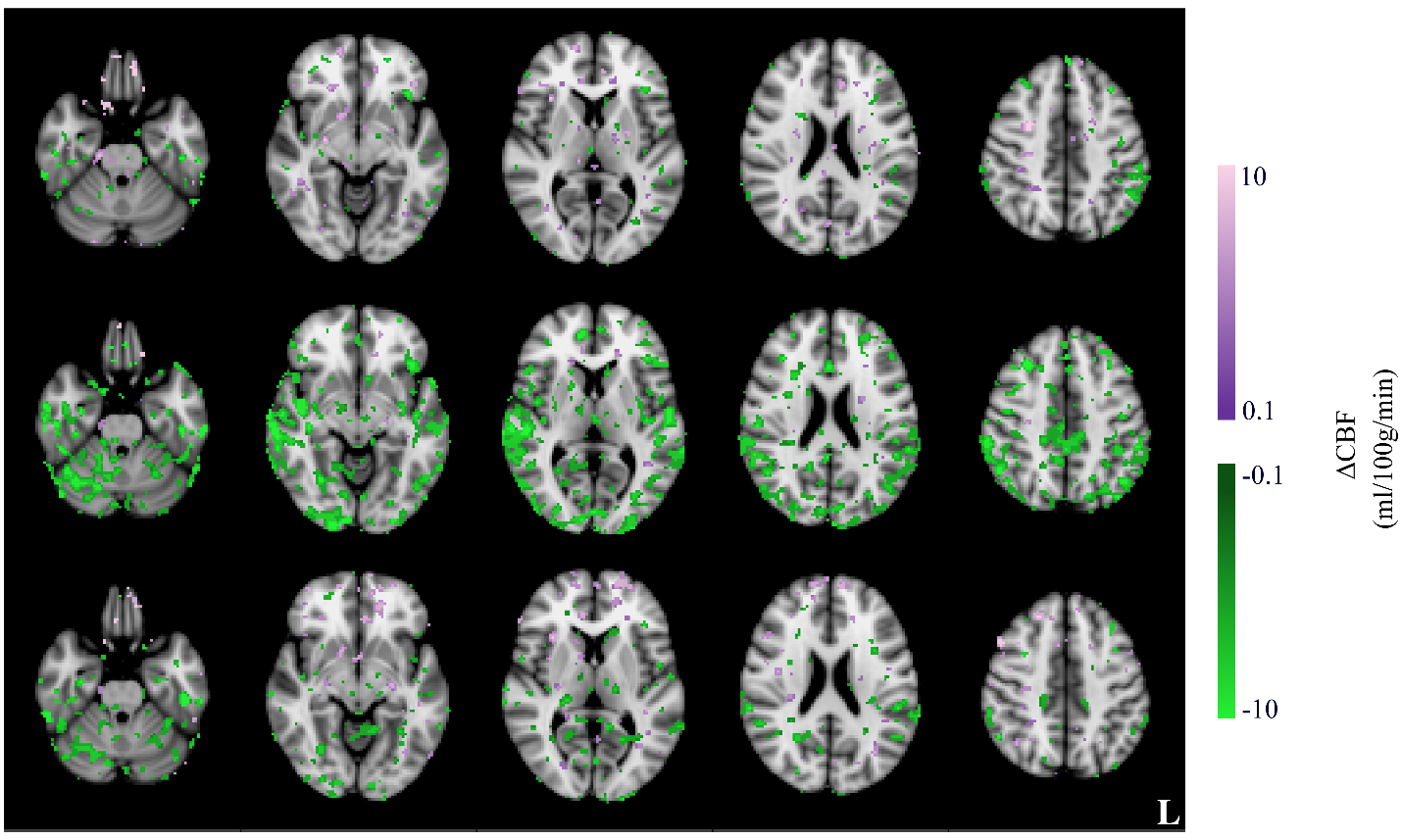


Supplemental Figure 4. ACT_1mA_ group-average changes in CBF for significant voxels from the *post hoc,* pairwise comparisons between session 1 pre-stimulation and (top row) session 1 post-stimulation, (middle row) session 2 post-stimulation and (bottom row) session 3 post-stimulation. Axial slices taken from MNI coordinates z = -26, -8, 6, 22, and 44mm (left to right).


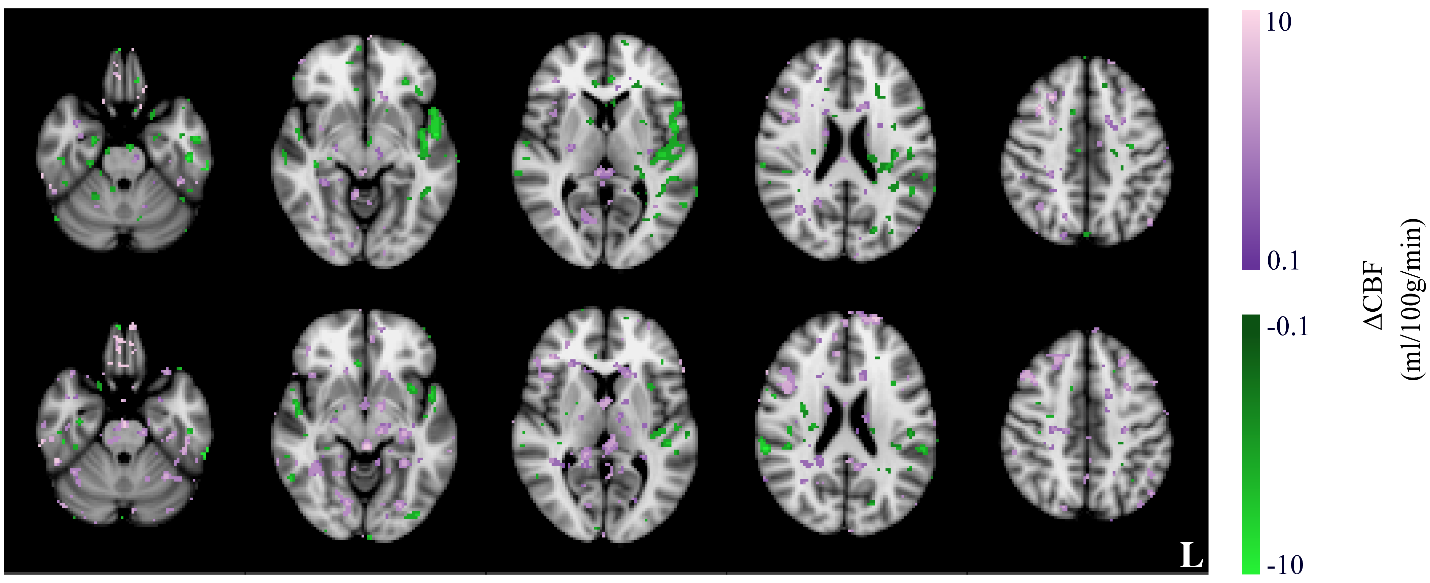


Supplemental Figure 5. ACT_2mA_ group-average changes in CBF for significant voxels from the *post hoc,* pairwise comparisons between session 1 pre-stimulation and (top row) session 1 post-stimulation, (middle row) session 2 post-stimulation and (bottom row) session 3 post-stimulation. Axial slices taken from MNI coordinates z = -26, -8, 6, 22, and 44mm (left to right).

Supplementary Table 1. Exclusion Criterion.

| Exclusion Criteria | |
| --- | --- |
| Neurological Disease or Diagnosis | Recent hospitalization for surgery/illness (within past 6 months) |
| Psychological Diagnosis/Hospitalization | Psychotropic Medications |
| Vision/Hearing Impairments that cannot be correct with vision or hearing aids | Any medication which may affect alertness |
| Problems with motor coordination | Shot (i.e., flu, allergies, pain) in the left arm (within last 4 weeks) |
| Use of implantable birth control device such as Implanon | Non-removeable metal or tattoos around head |
| Pregnant or could be pregnant | Color Blindness |
| Sleep Disorder | Tobacco Use |
| Drug or Alcohol treatment in last 6 months | Sensitivity to caffeine |
| Daily caffeine intake greater than 300mg per day | Alcohol consumption greater than 3 alcoholic beverages per day |
| Any metallic implants such as hearing aids, a pacemaker, artificial joints, plates, screws, or electrical devices | History of learning difficulty, frequent headaches, frequent eye strain, attention deficit, severe concussion or brain injury, recent head injury or mild concussion (past 30 days), seizures, fainting, migraines, high blood pressure, diabetes, heart disease |
| Claustrophobia |  |

Supplementary Table 2. Scan initiation times across subject and session. These times were taken from the header of the initial 3-plane localizer performed at the start of each scan.

|  |  | Session 1 | | Session 2 | | Session 3 | |
| --- | --- | --- | --- | --- | --- | --- | --- |
| Group | Subject # | Pre-tDCS | Post-tDCS | Pre-tDCS | Post-tDCS | Pre-tDCS | Post-tDCS |
| CON | 1 | 15:56:18 | 18:52:11 | 15:55:31 | 17:57:57 | 16:42:26 | 18:43:55 |
| CON | 2 | 17:32:36 | 20:02:56 | 17:27:17 | 19:59:25 | 13:46:19 | 15:46:39 |
| CON | 3 | 17:30:35 | 20:19:32 | 17:16:22 | 19:48:06 | 17:51:01 | 20:23:26 |
| CON | 4 | 17:48:06 | 20:20:09 | 17:33:40 | 20:23:03 | 16:20:00 | 19:05:17 |
| CON | 5 | 16:11:48 | 18:54:35 | 15:58:51 | 18:42:46 | 16:01:13 | 18:47:39 |
| CON | 6 | 16:23:18 | 18:23:31 | 17:16:31 | 19:15:41 | 17:03:40 | 18:58:49 |
| CON | 7 | 17:02:58 | 19:53:34 | 17:11:06 | 19:50:53 | 17:00:37 | 19:36:37 |
| CON | 8 | 19:14:00 | 21:58:09 | 19:16:31 | 21:45:02 | 18:47:15 | 21:15:16 |
| CON | 9 | 17:14:29 | 19:22:32 | 18:02:20 | 20:13:27 | 17:11:14 | 19:10:52 |
| CON | 10 | 17:25:20 | 20:10:12 | 17:06:18 | 19:35:13 | 17:22:45 | 19:58:13 |
| CON | 11 | 18:03:48 | 20:52:47 | 17:19:47 | 19:51:23 | 17:18:14 | 19:52:33 |
| CON | 12 | 18:50:41 | 21:39:55 | 18:22:04 | 20:53:11 | 18:39:27 | 21:17:52 |
| CON | 13 | 19:19:49 | 22:18:06 | 19:08:38 | 21:59:58 | 17:19:42 | 19:14:41 |
| CON | 14 | 17:17:02 | 20:09:10 | 17:01:22 | 19:45:38 | 17:04:54 | 19:38:02 |
| CON | 15 | 18:45:08 | 21:28:17 | 18:26:07 | 21:01:42 | 18:24:54 | 20:53:51 |
| ACT_1mA_ | 1 | 18:40:43 | 21:20:28 | 18:02:26 | 20:34:10 | 17:05:31 | 19:06:07 |
| ACT_1mA_ | 2 | 18:14:21 | 20:25:13 | 17:30:08 | 19:34:56 | 17:42:57 | 19:46:23 |
| ACT_1mA_ | 3 | 18:39:34 | 21:18:23 | 18:17:55 | 20:47:47 | 18:18:11 | 20:47:54 |
| ACT_1mA_ | 4 | 18:40:43 | 21:16:26 | 18:19:22 | 20:55:35 | 18:14:43 | 20:46:24 |
| ACT_1mA_ | 5 | 17:04:57 | 19:48:20 | 16:51:07 | 19:24:44 | 17:03:32 | 19:37:10 |
| ACT_1mA_ | 6 | 18:27:53 | 21:07:31 | 18:07:27 | 20:40:07 | 18:17:28 | 20:51:35 |
| ACT_1mA_ | 7 | 17:22:00 | 20:13:17 | 17:01:51 | 19:36:44 | 17:28:24 | 19:59:26 |
| ACT_1mA_ | 8 | 19:03:19 | 21:49:40 | 18:51:04 | 21:22:58 | 18:33:07 | 21:12:08 |
| ACT_1mA_ | 9 | 19:05:06 | 21:40:44 | 18:21:56 | 20:52:31 | 18:22:37 | 20:54:57 |
| ACT_1mA_ | 10 | 18:31:41 | 21:05:10 | 18:55:26 | 21:30:34 | 18:18:55 | 20:47:30 |
| ACT_1mA_ | 11 | 17:36:19 | 20:28:40 | 17:19:11 | 19:58:10 | 17:34:05 | 20:13:35 |
| ACT_1mA_ | 12 | 17:22:17 | 20:25:30 | 17:06:10 | 19:38:14 | 17:15:02 | 19:59:56 |
| ACT_1mA_ | 13 | 17:31:17 | 19:39:00 | 17:14:56 | 19:25:00 | 17:22:54 | 19:28:48 |
| ACT_1mA_ | 14 | 18:18:24 | 21:19:10 | 17:55:57 | 20:27:13 | 17:17:40 | 20:13:16 |
| ACT_1mA_ | 15 | 18:52:48 | 21:34:02 | 18:24:37 | 20:59:02 | 18:26:39 | 21:09:49 |
| ACT_2mA_ | 1 | 15:27:16 | 18:01:59 | 14:57:15 | 17:28:32 | 15:18:50 | 17:51:01 |
| ACT_2mA_ | 2 | 16:44:11 | 19:21:04 | 16:13:38 | 18:44:05 | 16:35:12 | 19:06:42 |
| ACT_2mA_ | 3 | 16:06:48 | 18:58:18 | 15:57:57 | 18:31:32 | 16:34:38 | 19:07:10 |
| ACT_2 mA_ | 4 | 17:11:41 | 19:36:09 | 17:21:46 | 19:20:47 | 18:33:52 | 20:32:34 |
| ACT_2 mA_ | 5 | 16:26:46 | 19:03:58 | 16:18:45 | 19:07:40 | 17:50:30 | 20:21:49 |
| ACT_2 mA_ | 6 | 17:33:52 | 20:17:01 | 17:22:00 | 20:05:47 | 17:30:17 | 20:06:59 |
| ACT_2 mA_ | 7 | 17:08:27 | 19:17:56 | 16:58:05 | 19:04:03 | 17:16:55 | 19:21:15 |
| ACT_2 mA_ | 8 | 18:27:45 | 21:27:10 | 18:28:02 | 21:12:19 | 18:19:03 | 20:54:40 |
| ACT_2 mA_ | 9 | 17:55:16 | 20:43:52 | 17:48:03 | 20:30:38 | 17:31:46 | 20:01:43 |
| ACT_2 mA_ | 10 | 19:02:28 | 21:42:55 | 17:09:04 | 19:08:06 | 17:12:30 | 19:15:45 |
| ACT_2 mA_ | 11 | 17:09:15 | 19:50:32 | 17:33:47 | 20:12:17 | 17:04:35 | 19:32:37 |
| ACT_2 mA_ | 12 | 19:27:56 | 22:26:39 | 18:35:12 | 21:06:37 | 18:35:46 | 21:07:21 |
| ACT_2 mA_ | 13 | 19:02:28 | 22:21:26 | 18:40:13 | 21:16:50 | 18:50:00 | 21:30:50 |
| ACT_2 mA_ | 14 | 18:11:10 | 20:23:41 | 17:22:14 | 19:55:06 | 17:19:27 | 19:40:07 |
| ACT_2 mA_ | 15 | 18:53:36 | 21:41:46 | 18:34:54 | 21:09:48 | 18:35:57 | 21:14:22 |
| ACT_2 mA_ | 16 | 17:28:16 | 20:20:42 | 17:14:55 | 19:50:26 | 17:15:37 | 19:57:34 |
| ACT_2 mA_ | 17 | 17:28:51 | 20:14:47 | 17:06:19 | 19:40:19 | 17:09:49 | 19:41:41 |
